# The impact of different pre-operative interventional hair- removal behaviours on biophysical characteristics and surgical site infection –a comparative analysis

**DOI:** 10.1017/ash.2025.106

**Published:** 2025-09-03

**Authors:** Veena Kumari, Ann Devapriya, Mariamma Philip, Muralidharan K, Shashank Devapur Vasant

**Affiliations:** 1 Professor & ICO, Department of NeuromicrobiologyNIMHANS, Bengaluru, India; 2 Sr. Nursing Officer & ICN, Hospital Infection Surveillance System (HISS), Dept of NeuromicrobiologyNIMHANS, Bengaluru, India; 3 Addl. Professor, Dept of BiostatisticsNIMHANS, Bengaluru, India; 4 Professor & Medical Supt, Dept of PsychiatryNIMHANS, Bengaluru, India; 5 Inflexon Academy (A unit of IHS LLP), Bengaluru, Karnataka, India

**Keywords:** Surgical Clipper, Razor, SSI, Pre-operative, Neurosurgery

## Abstract

**Introduction:** Razors were being used for pre-operative hair removal in our Institute. As per international guidelines recommending the use of surgical clippers, we opted to study the effects of two pre-operative skin preparations in our Neurosurgical centre **Objectives:** **Primary; Pre auditing period** -Assess knowledge and skill in usage of Razor/Clipper as preoperative skin preparation methods, Provide **training** on Clipper method and assess the knowledge /awareness on merits and demerits of both methods**, To implement** the Clipper method as against Shaving **Secondary ; Post auditing period** -Investigate the efficacy and safety of clippers versus razors, on variety of biophysical parameters and Surgical Site Infection (SSI) **Methods :** PICO questions ; Population: Adult patients undergoing any type of surgical procedure, Intervention: Hair removal, Comparator: Different methods of Hair removal, Outcomes: Biophysical parameters and SSI •**Target population: Sixty adult patients** undergoing neurosurgical procedures. •**Subjects:** 30 each subjected to shaving and clipper methods •Pre and Post assessment of on **25 parameters** /sub-parameters •Analysis by MS-Excel and SPSS. **Results: •Preoperative** -Prior skin injuries and/or reactions; adequacy of hair removal •**Complete hair removal** : 30 (100%) in the clipper group versus 3 (10%) by shaving **(p = 0.0001).** •**30 mins after hair removal** ; significantly less skin issues in the clipper group •**Post operative** - Skin injuries in 20 (66.6%) of the razor and none in the clipper group. •**SSI** - Two **(6.6%) in the razor** and none in the clipper group. **Conclusions:** The assessment showed that shaving leads to partial hair removal increasing the scores for skin issues, significant association between preoperative skin injuries and SSI, implying inverse correlation with the clipper method. This study provides insights into significance of among other biophysical parameters underscoring adoption of clipper as the standard practice for preoperative hair removal, in our setting thus enhancing patient safety.

